# Measurement of market (industry) concentration based on value validity

**DOI:** 10.1371/journal.pone.0264613

**Published:** 2022-07-18

**Authors:** Tarald O. Kvålseth

**Affiliations:** Department of Mechanical Engineering and Department of Industrial & Systems Engineering, University of Minnesota, Minneapolis, MN, United States of America; AGROCAMPUS OUEST - Centre d’Angers, FRANCE

## Abstract

As measures of concentration, especially for market (industry) concentration based on market shares, a variety of different measures or indices have been proposed. However, the various indices, including the two most widely used ones, the concentration ratio and the Herfindahl-Hirschman index (*HHI*), lack an important property: the value-validity property. An alternative index with this and other desirable properties is introduced. The new index makes it permissible to properly assess the extent of the concentration and make order and difference comparisons between index values as being true representations of the real concentration characteristic (attribute). Computer simulation data and real market-share data are used in the analysis. It is shown that the new index has a close functional relationship with the *HHI* index and has a firm theoretical relationship with market power as measured by the price-cost margin. Corresponding modifications to existing merger guidelines are presented.

## 1. Introduction

When dealing with nominal data, as with all types of data, it is often of interest to use some summary measures to represent certain characteristics or attributes associated with the data. One such characteristic is the concentration reflected by the probabilities or proportions *p*_1_,…,*p*_*n*_ of *n* mutually exclusive and exhaustive categories or events. Concentration is then considered high when one or a few of the *p*_*i*_‘s are relatively large and decreases as the *p*_*i*_‘s become increasingly equal. The converse characteristic, qualitative variation (evenness), is measured by indices whose values increase as the *p*_*i*_‘s become increasingly even, equal, or uniform (e.g., [1, 2]).

One area in which the measurement of concentration has a long history is that of measuring market concentration or industrial concentration where the *p*_*i*_ (*i* = 1,…,*n*) are the market shares of the *n* firms within a market or industry (e.g., [3, Ch. 4], [4, 5, pp. 221–238], [6, Ch. 8], [7–9]). Increasing market concentration tends to decrease competition and efficiency and increase market power. Any such trends are of concern to the business community and are being monitored by the U.S. Department of Justice (DOJ) and the Federal Trade Commission (FTC) in the case of antitrust. While such potentially negative consequences from increasing market concentration is the generally accepted proposition, it does not necessarily apply universally. In fact, a number of empirically-based exceptions have been documented and reported [7, 10–12]. However, no one disputes the fact that concentration is an important market indicator.

A number of different indices or measures of market (industry) concentration have been proposed over the years as outlined in this paper. However, two such indices have become by far the most popular ones: the *m-firm concentration ratio* and the *Herfindahl-Hirschman* index *HHI* (e.g., [13, pp. 116–118], [14, pp. 97–101], [6, Ch. 8], [15]). The *m-firm concentration ratio* (*CR*_*m*_) is simply defined as the sum of the market shares of the *m* largest firms in a market, with the 4-firm *CR*_*4*_ being the most commonly used member of the *CR*_*m*_ *-*family [16, p. 255]. The *HHI*, after Herfindahl [17] and Hirschman [18], includes all market shares and is defined as the sum of their squares. While *CR*_*4*_ was used in the earliest 1968 *Merger Guidelines* by the DOJ and FTC [19], the later guidelines, with the most recent being the 2010 *Horizontal Merger Guidelines* [19], have been using the *HHI* as a screening tool for potential antitrust concerns from mergers. The preference for *HHI* over *CR*_*4*_, both for practical screening and public policy as well as for research and analysis, is based on the much more comprehensive form of *HHI* (e.g., [7]).

This paper points out a serious limitation of the various concentration indices, including *CR*_*m*_ and *HHI*, that is specifically related to the numerical values taken on by the indices for different market-share distributions. While those indices have some highly desirable mathematical properties, no concern seems to have been raised so far about an important question concerning the actual values of an index: does a concentration index *C* take on numerical values throughout its range that can be rigorously explained or justified as providing realistic, true, or valid representations of the concentration characteristic or attribute? That is, does *C* have *value validity*?

A reason for this concern is the fact that different indices with similar mathematical properties can produce wildly different values for the same data sets. Comparisons between index values can therefore result in potentially misleading and unreliable interpretations, findings, and conclusions. What is needed of a concentration index C is an additional property requirement and its basic condition to provide C with value validity. This is the focus of this paper.

This paper is organized as follows. First, the set of properties generally required of a concentration index *C* are defined and discussed, followed by a definition and discussion of the *value-validity property* of *C*. Second, a concise historical account is given of the various indices that have been proposed over the years and their lacking properties are identified, with particular emphasis on the two indices *CR*_*4*_ and *HHI*. Third, since existing indices lack the value-validity property, a new index *C*_*K*_ with this and other necessary properties is introduced and discussed as an extension of the author’s earlier and preliminary effort to measure the homogeneity of categorical data [20]. Fourth, *C*_*K*_ is compared with other indices, notably *CR*_*4*_ and more so *HHI*, using both computer simulation data and real market-share data. Fifth, an approximate functional relationship between *C*_*K*_ and *HHI* is established with impressive accuracy and serving various pruposes: (i) the behavior and changes in *HHI* can be compared with those of *C*_*K*_, (ii) reported values of *HHI* can be converted to those of *C*_*K*_, (iii) the theoretical relationship between industry profitability and *HHI* can be extended to that of *C*_*K*_, and (iv) the DOJ and FTC merger guidelines based on *HHI* can be equivalently expressed in terms of *C*_*K*_.

Since the new index *C*_*K*_ has the value-validity property as proved in this paper, *C*_*K*_ is equally sensitive to changes in a market-share distribution throughout its potential range of values, permitting order and difference comparisons between index values. By contrast, other indices such as the most widely used *HHI* lack this property so that different types of comparisons between index values reflect those of an index itself rather than of the concentration attribute being measured. In the case of *HHI*, the effect of lacking the value-validity property implies that *HHI* lacks adequate sensitivity to changes in the market-share distribution for smaller values of *HHI* whereas it has excessive sensitivity for larger values of *HHI*. This important difference between *C*_*K*_ other indices, notably *HHI*, is indeed relevant when considering the anticompetitive effects of mergers and the ongoing debate about whether or not and to what extent concentration and market power have increased over the recent decades (e.g., [21–24]).

## 2. Required properties

### 2.1 General requirements

In order for *C* to qualify as a concentration index, it has to have a number of specific properties as discussed extensively in the published literature (e.g., [3, pp.47-58] [4, 25, 26]). First, however, a comment on notation: while the strictly mathematically correct notation would be to refer to *C*(*P*_*n*_) as being the value of the index or function *C* for the distribution *P*_*n*_ = (*p*_1_,…,*p*_*n*_), *C* and *C*(*P*_*n*_) will both be used throughout this paper to denote both an index (function) and its value in order to simplify the notation when there is no chance of ambiguity. Required properties of *C* may then be outlined as follows:

(P1) *Continuity*: *C* is a continuous function of all the *p*_*i*_ (*i* = 1,…,*n*).
(P2) *Symmetry*: *C* is (permutation) symmetric in *p*_1_,…,*p*_*n*_.
(P3) *Zero-indifference*: *C* is unaffected if one or more firms with zero market share enter the market, i.e., *C*(*p*_1_,…,*p*_*n*_, 0…,0) = *C*(*p*_1_,…,*p*_*n*_).
(P4) *Schur-convexity*: *C* is strictly Schur-convex.
(P5) *Value validity*: C has value validity.

The continuity Property (P1) ensures that small changes in some of the *p*_*i*_ (*i* = 1,…,*n*) result in only a small change in the value of *C*. Property (P2) simply states that *C* is invariant with respect to the order in which the original *p*_*i*_ (*i* = 1,…,*n*) are given (e.g., [27], [5, p. 222]). According to Property (P3), the addition or deletion of one or more *p*_*i*_ = 0 components to or from the distribution *P*_*n*_ = (*p*_*i*_,…,*p*_*n*_) has no effect on the value of *C*. Property (P3) has two important implications. First, with respect to market concentration, (P3) together with (P1) and (P4), has the effect of slightly decreasing the value of *C* if one or more additional small firms enter the market (or the reverse effect if the small firms leave the market) (e.g., [28, Ch. 8], [3, p. 53]). Second, Property (P3) implies that *C* cannot be an explicit function of *n* such as if *C* were to be normed to some specific range by adjusting (controlling) for *n*. Properties (P4)-(P5) are subsequently defined and discussed in detail.

Another property advocated by some is the so-called replication property. First suggested by Hall and Tideman [29], this property means that if each *p*_*i*_ is split into *k* equal parts *p*_*i*_/*k*,…,*p*_*i*_/*k* for *i* = 1,…,*n*, resulting in the *kn* component distribution denoted by *P*_*n*_/*k*, then *C* should be reduced by a factor of 1/*k*, i.e., *C*(*P*_*n*_/*k*) = *C*(*P*_*n*_)/*k*. Replication, which is different from the homogenous property of a function, may be an interesting and novel mathematical concept, but is entirely unrealistic of any practical situation involving concentration. While favored by some (e.g., [29, 30]), others do not view replication as a necessary property (e.g., [3, pp.47-58]), some do not even mention replication among the properties of a concentration index (e.g., [5, pp.221-223], [27]), and some feel that it is not self-evident that this property is desirable [31, pp. 63–64]. The index *C* and its normalized form C*∈[0, 1] cannot both have the replication property, but no rigorous explanation has been offered as to which one should have this property and why. It is also worth noting that the (sample) standard deviation *s*_*n*_ (with devisor *n*) of *p*_1_,…,*p*_*n*_ has the replication property, but *s*_*n*−1_ (with devisor *n*-1) does not (nor does the variance $s_{n}^{2}$). However, nobody would argue that *s*_*n*_ is to be preferred over *s*_*n*−1_ because of the replication principle even though *s*_*n*−1_ has the statistical property of unbiasedness, nor would *s*_*n*_ be preferred over $s_{n}^{2}$ for this reason.

Most importantly, however, is the fact that no generally accepted and rigorous basis appears to have been provided for the replication property as being of any importance to a concentration index. Also, from the value-validity property (P5) discussed below, it becomes apparent that replication is inconsistent with this property. It appears that the replication property lacks any real or solid justification.

### 2.2 Schur-convexity

The strict Schur-convexity Property (P4), which was also discussed by Hannah and Kay [3, Ch. 4], means that if the components of *P*_*n*_ = (*p*_1_,…,*p*_*n*_) are “less unevenly distributed” or “less spread out” than are those of another distribution *Q*_*n*_ = (*q*_1_,…,*q*_*n*_), then *C*(*P*_*n*_)<*C*(*Q*_*n*_). Such rather vague statements can be expressed more precisely in terms of *majorization theory* (e.g., Marshall et al. [32]). Thus, by definition, if the *p*_*i*_‘s are ordered such that

$$p_{1}\geq p_{2}\geq \cdots \geq p_{n}$$ (1)

then *P*_*n*_ is *majorized by Q*_*n*_ (denoted by *P*_*n*_≺*Q*_*n*_) under the following condition:

$$P_{n}≺Q_{n}\;if\;\sum_{i=1}^{j}p_{i}\leq \sum_{i=1}^{j}q_{i},\;j=1,\ldots ,n-1$$ (2)

with $\sum_{i=1}^{n}p_{i}=\sum_{i=1}^{n}q_{i}=1$. *C* is then strictly Schur-convex if

$$P_{n}≺Q_{n}\;\mathrm{implies}\;C(P_{n})<C(Q_{n})$$ (3)

assuming *P*_*n*_ is not a permutation of *Q*_*n*_ [32, pp. 8, 80]. If the inequality in (3) is not strict, then *C* is Schur-convex.

The condition in (3), which is referred to by economists as the *Dalton condition* or the *Pigou-Dalton condition*, also implies that (a) *C* has the *transfer property* and (b) *C* preserves the *Lorenz order* [32, pp. 5–8, 560, 712–723]. The principle of “upward” transfers means that if *p*_*i*_>*p*_*j*_ and an amount *δ*<*p*_*i*_−*p*_*j*_ is transferred from *p*_*j*_ to *p*_*i*_, the value of *C* increases. The fact that the strictly Schur-convex *C* preserves the Lorenz order means that if the distribution *P*_*n*_ is majorized by *Q*_*n*_ as defined in (2), then (a) the Lorenz curve (after [33]) for *P*_*n*_ falls nowhere below the Lorenz curve for *Q*_*n*_ and (b) *C*(*P*_*n*_)<*C*(*Q*_*n*_). By definition, if the *p*_*i*_‘s are ordered such that *p*_(1)_≤*p*_(2)_≤…≤*p*_(*n*)_, with $F_{\left(i\right)}=\sum_{j=1}^{i}p_{\left(j\right)}$ and *F*_(0)_ = 0, then the Lorenz curve for *P*_*n*_ is obtained from the line segments between the consecutive points (*i*/*n*, *F*_(*i*)_ for *i* = 0,…,*n*.

### 2.3 Value validity

#### 2.3.1 The importance of the value-validity property

In order to provide a simple confirmation of the need to impose a restriction on the values of a concentration index, consider first the complete lack of uniformity among the values taken on by different concentration indices for the same data sets. For some of the indices *C*_*i*_ (defined and discussed in Section 3 below) and for the distribution $P_{10}^{0.5}=(0.55,\;0.05,\ldots ,\;0.05)$, the respective values of the indices *C*_1_(*CR*_4_), *C*_3_(*HHI*), *C*_4_, *C*_6_, *C*_8_, *C*_9_, *C*_13_, *C*_14_, and *C*_17_ become 0.70, 0.33, 0.18, 0.59, 0.48, 0.41, 0.19, 0.14, and 0.45. That is as much as 400% variation in index values for the same data set in spite of the fact that many of these indices have the same properties. Even the values of *C*_3_(*HHI*) and *C*_13_ differ by a factor of nearly 2 although both indices are members of the parameterized family *C*_*18*_ (with *α* = 1 and *α*→0).

In fact, one could define a family of indices as the power function (*HHI*)^*α*^ with arbitrary parameter *α*>0 and with all members having Properties (P1)-(P4). Of course, the values of different family members could vary greatly for the same data set and difference comparisons between index values for different data sets could vary greatly, depending upon *α*. The need for some additional constraint on index values would seem to be paramount.

Such variation in index values is also reflected in their differing sensitivities to changes in the distribution *P*_*n*_ = (*p*_1_,…,*p*_*n*_). In the case of the most important index *HHI*, as discussed more extensively later in the paper, *HHI* is more sensitive to changes in *P*_*n*_ for large index values than for small index values. As a numerical illustration, consider the two distributions (0.30, 0.30, 0.20, 0.20) and (0.35, 0.30, 0.20, 0.15) for which *HHI* = 0.26 and 0.28, respectively, where the first distribution is obtained from the second one by means of a transfer as defined above. Then, consider the same transfer involving the two distributions (0.70, 0.15, 0.10, 0.05) and (0.75, 0.15, 0.10, 0.00) for which *HHI* = 0.53 and 0.60. The increase in *HHI* values between the last two more concentrated distributions is about three times that between the first two less concentrated distributions (i.e., 0.07 versus 0.02), with relative increases of 13.2% and 7.7%.

Since all proposed concentration indices take on their extremal values for the two distributions

$$P_{n}^{0}=(\frac{1}{n},\ldots ,\frac{1}{n}),\;P_{n}^{1}=(1,0,\ldots ,0),$$ (4)

it may be interesting and informative to consider index values for the following mean of $P_{n}^{0}$ and $P_{n}^{1}$:

$$P_{n}^{0.5}=(\frac{1}{2})P_{n}^{0}+(\frac{1}{2})P_{n}^{1}=(\frac{1}{2}+\frac{1}{2n},\frac{1}{2n},\ldots ,\frac{1}{2n}).$$ (5)

In terms of this information alone, the only defensible value of a concentration index *C* is that its value for $P_{n}^{0.5}$ in (5) should be the mean of its values for $P_{n}^{0}$ and $P_{n}^{1}$ in (4), i.e.,

$$C(P_{n}^{0.5})=(\frac{1}{2})C(P_{n}^{0})+(\frac{1}{2})C(P_{n}^{1}).$$ (6)

For the $P_{10}^{0.5}=(0.55,\;0.05,\ldots ,\;0.05)$ used in the above example and with $C(P_{n}^{0})=1/n$ and $C(P_{n}^{1})=1$ as for many indices, including *HHI*, (6) requires that $C(P_{10}^{0.5})=0.55$. However, by comparison, $HHI(P_{10}^{0.5})=0.33$ i.e., 40% less than the corresponding requirement from (6). Other indices fare no better, such as $C_{14}(P_{10}^{0.5})=0.14<<0.55$.

The requirement in (6) can also be expressed in terms of distances or differences as

$$C(P_{n}^{1})-C(P_{n}^{0.5})=C(P_{n}^{0.5})-C(P_{n}^{0})$$ (7)

showing the $C(P_{n}^{0.5})$ is an equal distance from each of the two extreme values $C(P_{n}^{0})$ and $C(P_{n}^{1})$. Furthermore, by considering the distributions $P_{n}^{0},\;P_{n}^{0.5},\;\mathrm{and}\;P_{n}^{1}$ as being points or vectors in *n*-dimensional Euclidean space, the following equality between Euclidean distances can be seen to hold:

$$d(P_{n}^{1},P_{n}^{0.5})=d(P_{n}^{0.5},P_{n}^{0}).$$ (8)

That is, the distance between $P_{n}^{0.5}\;\mathrm{and}\;P_{n}^{1}$ is the same as that between $P_{n}^{0.5}$ and $P_{n}^{0}$, supporting the requirement in (7).

#### 2.3.2 Generalized requirement

The development from (4) to (8) can be further generalized by using the *lambda distribution*

$$P_{n}^{\lambda }=(\lambda +\frac{1-\lambda }{n},\;\frac{1-\lambda }{n},\ldots ,\frac{1-\lambda }{n}),\;0\leq \lambda \leq 1$$ (9)

introduced by Kvålseth [34] (the 1−*λ* is used here as a convenient form when dealing with concentration). This particular distribution provides an important and general basis for the value-validity property as first discussed in Kvålseth [34]. The present paper adapts those basic concepts to the measurement of concentration.

The real-valued parameter *λ* is basically a concentration parameter of which *λ* = 0, *λ* = 0.5, and *λ* = 1 are the particular cases in (4) and (5). In fact, the following linear function or weighted arithmetic mean of $P_{n}^{0}$ and $P_{n}^{1}$

$$P_{n}^{\lambda }=(1-\lambda )P_{n}^{0}+\lambda P_{n}^{1},\;0\leq \lambda \leq 1$$ (10)

includes the mean in (5) as a particular case with *λ* = 1/2. Furthermore, the condition in (6) would be a particular case of the general formulation

$$C(P_{n}^{\lambda })=(1-\lambda )C(P_{n}^{0})+\lambda C(P_{n}^{1}).$$ (11)

Similarly, it is seen that (7) and (8) generalize as follows:

$$C(P_{n}^{1})-C(P_{n}^{\lambda })=(\frac{1-\lambda }{\lambda })[C(P_{n}^{\lambda })-C(P_{n}^{0})]$$ (12)

and

$$d(P_{n}^{1},P_{n}^{\lambda })=(\frac{1-\lambda }{\lambda })d(P_{n}^{\lambda },P_{n}^{0}).$$ (13)

Besides considering (11) as a direct extension of (6), although clearly supported by (12)-(13), the general condition for value validity in (11) also follows from the requirement that *C* should be equally sensitive to small changes in the distribution *P*_*n*_ = (*p*_1_,…,*p*_*n*_) throughout its range from $C(P_{n}^{0})$ to $C(P_{n}^{1})$. This sensitivity requirement can equivalently be expressed in terms of the *discriminant ability* of *C*, i.e., the ability of *C* to discriminate between distributions *P*_*n*_ that are not unduly different. Thus, *C* should have a constant or uniform ability to detect small changes in any *P*_*n*_. Such a requirement can be imposed on an index *C* with Properties (P1)-(P4) because of the following equality:

$$C(P_{n})=C(P_{n}^{\lambda })\;\mathrm{for}\;\mathrm{one}\;\mathrm{unique}\;\lambda$$ (14)

for any *P*_*n*_ and the $P_{n}^{\lambda }$ in (10). Consequently, as the *p*_*i*_‘s become increasingly unequal or uneven so that *C*(*P*_*n*_) increases, the value of the concentration parameter *λ* increases correspondingly for any given *n*. As a simple illustration involving the index *HHI*, consider *HHI*(0.50, 0.30, 0.20) = 0.38 and *HHI*(0.70, 0.20, 0.10) = 0.54 for which the respective values of *λ* from (14) is found to be *λ* = 0.26 and *λ* = 0.56.

The requirement or condition that *C*(*P*_*n*_) should be equally sensitive to small changes in the form of *P*_*n*_ throughout its range becomes equivalent to requiring the partial derivative $\partial C(P_{n}^{\lambda })/\partial \lambda$ to be constant for all *λ* and any given *n*. Consequently, $C(P_{n}^{\lambda })$ has to be a linear function of *λ*, which leads immediately to (11).

## 3. Assessment of indices

A concise historical account of concentration indices proposed to date is summarized in Table 1. Some of these are parameterized families of indices such as *C*_10_, *C*_11_, *C*_12_, *C*_18_, and *C*_19_ since they depend on an arbitrary parameter *α*. Two of the individual indices, C_3_(*HHI*) and *C*_*13*_, are seen to be members of the *C*_*18*_-family (for *α* = 1 and *α*→0). Note that, although all indices in Table 1 have the Symmetry Property (P2), the indices *C*_1_, *C*_4_, *C*_6_, *C*_7_, *C*_9_, and *C*_17_ are based on the descending order among the *p*_*i*_‘s as in (1).

**Table 1:** Proposed concentration indices based on probabilities (proportions, market shares) *p*_1_≥*p*_2_≥⋯≥*p*_*n*_ and their lacking properties (LP, in Section 2.1).

*C*_*i*_	Formula	Reference	Notes	LP
*C*_1_ (*CR*_*m*_)	$\sum_{i=1}^{m}p_{i}$	Various authors		P1, P4
*C*_2_	$\sqrt{\sum_{i=1}^{n}p_{i}^{2}}$	Hirschman [18]		P5
*C*_3_ (*HHI*)	$\sum_{i=1}^{n}p_{i}^{2}$	Herfindahl [17]		P5
*C*_4_	${\left(2\sum_{i=1}^{n}ip_{i}-1\right)}^{-1}$	Rosenbluth [25] and Hall & Tideman [29]		P5
*C*_5_	$‐\sum_{i=1}^{n}p_{i}\log p_{i}$	Theil [28]	a	P5
*C*_6_	$p_{1}+\sum_{i=2}^{n}p_{i}^{2}(2-p_{i})$	Horvath [35]		P4, P5
*C*_7_	$\sum_{i=1}^{m}p_{i}(m+1-i)/m,\;m\geq 1$	Hart [36]	b	P1, P3, P4
*C*_8_	$2\sum_{i=1}^{n}p_{i}^{2}-\sum_{i=1}^{n}p_{i}^{3}$	Hart [37]	c	P5
*C*_9_	$\frac{1}{n(n-1)}\sum_{m=1}^{n-1}\left(\frac{n-m}{m}\right)(\frac{CR_{m}}{1-CR_{m}})$	Linda [38]		P1,P3,P4,P5
*C*_10_	$\sum_{i=1}^{n}\left[p_{i}^{2}+{\left\{p_{i}(\sum_{j=1}^{n}p_{j}^{2}-p_{i}^{2})\right\}}^{\alpha }\right]$, *α*>1	Hause [39]		P5
*C*_11_	${\left(\sum_{i=1}^{n}p_{i}^{\alpha }\right)}^{1/(1-\alpha )}$, *α*>0	Hannah & Kay [3]	d	P5
*C*_12_	${\left(n\sum_{i=1}^{n}p_{i}^{2}\right)}^{\alpha }/n,\;\alpha >0$	Davies [40]		P1, P3, P5
*C*_13_	$\prod_{i=1}^{n}p_{i}^{p_{i}}$	Bruckmann [41], Häni [42]		P5
*C*_14_	$\sum_{i=1}^{n}p_{i}{\left[1+n(1-p_{i})\right]}^{-1}$	Ginevičius [43]		P1, P3, P5
*C*_15_	$\sum_{i=1}^{m}p_{i}^{2}/\sum_{i=1}^{n}p_{i}^{2},\;m\geq 1$	Anbarci & Katzman [44]		P4, P5
*C*_16_	$\sum_{i=1}^{n}{\left(\log p_{i}\right)}^{2}/n-{\left(\sum_{i-1}^{n}\log p_{i}\right)}^{2}/n^{2}$	Hannah & Kay [3]		P1, P4, P5
*C*_17_	$1-\left(2\sum_{i=1}^{n}ip_{i}-1\right)/n$	Marfels [45]		P1, P3
*C*_18_	${\left(\sum_{i=1}^{n}p_{i}{}^{\alpha +1}\right)}^{1/\alpha },\;\alpha >-1$	Bruckmann [41]		P5
*C*_19_	$\alpha^{-1}\log\sum_{i=1}^{n}p_{i}e^{\alpha p_{i}},\;\alpha >0$	Bruckmann [41]		P5

Notes: (a) This is the famous entropy by Shannon [46], it is strictly Schur-concave; (b) Hart [36] also suggested replacing *m* in *C*_7_ with *n*; (c) The *C*_8_ was proposed as an alternative to *C*_6_; (d) This family of indices ranges from $C_{11}(P_{n}^{0})=n\;\mathrm{to}\;C_{11}(P_{n}^{1})=1$ for the market-share distributions in (4) and measures deconcentration; it is strictly Schur-concave.

As indicated in the right column of Table 1, the various indices lack some of the Properties (P1)-(P5) as can easily be verified. A number of the potential indices lack the strict Schur-convexity (Property (P4)) while several others lack the zero-indifference Property (P3). Only three of the indices, *C*_1_(*CR*_*m*_), *C*_7_, and *C*_17_, meet the condition in (11) required by the value-validity Property (P5). The other indices fail to meet even the weaker condition in (6). However, each of those three indices lack other properties as indentified in Table 1. In particular, *CR*_*m*_ lacks Property (P1) and the strict Schur-convexity (Property (P4)), although it can be shown to be Schur-convex.

In the case of *C*_3_(*HHI*) and the distributions of $P_{n}^{0}\;\mathrm{and}\;P_{n}^{1}$ in (4) and $P_{n}^{\lambda }$ in (9), it is seen that

$$HHI(P_{n}^{\lambda })=\lambda^{2}HHI(P_{n}^{1})+(1-\lambda^{2})HHI(P_{n}^{0})=(1-\frac{1}{n})\lambda^{2}+\frac{1}{n}$$ (15)

with $HHI(P_{n}^{0})=1/n$ and $HHI(P_{n}^{1})=1$. When compared with (1−1/*n*)*λ*+1/*n* as required by (11), it is clear that *HHI* understates the true extent of the concentration. This negative bias occurs throughout the range of values of *HHI*, but is seen to be most pronounced when *λ* = 1/2. From the partial derivative $\partial HHI(P_{n}^{\lambda })/\lambda =2(1-1/n)\lambda$ and the equality in (14), the implication is that for any given *n*, *HHI*(*P*_*n*_) is not equally sensitive to small changes in *P*_*n*_ throughout its range. Rather, *HHI*(*P*_*n*_) has the bias of being increasingly sensitive to changes in *P*_*n*_ = (*p*_1_,…,*p*_*n*_) as the *p*_*i*_‘s become increasingly uneven or variable, i.e., with increasing *HHI*(*P*_*n*_)-values. The important point is this: while a concentration index should clearly be sensitive to changes in *P*_*n*_, such sensitivity should not be biased in a particular direction such as toward uneven (skewed) distributions as in the case of *HHI*.

## 4. The new index

### 4.1 Index formulation

Several of the indices defined in Table 1 can be considered as members of the following class of weighted sums:

$$C=\sum_{i=1}^{n}w_{i}p_{i}$$ (16)

where *w*_1_,…,*w*_*n*_ are positive weights with each *w*_*i*_ being a function of (or depending upon) *p*_*i*_ and possibly other components of the distribution *P*_*n*_ = (*p*_1_,…,*p*_*n*_). Other indices in Table 1 can be viewed as strictly increasing functions of the *C* in (16). The most obvious members of (16) are perhaps *HHI* with *w*_*i*_ = *p*_*i*_ for *i* = 1,…,*n* and *CR*_*m*_ with *w*_*i*_ = 1 for *i* = 1,…,*m* and 0 otherwise (with the *p*_*i*_’*s* being ordered as in (1)).

A family of concentration indices as in (16) can also be derived from theoretical models. For example, Encaoua and Jacquemin [27] arrived at (16) by means of an axiomatic characterization. In their derivation, *w*_*i*_ is a nondecreasing function of *p*_*i*_ such that *w*_*i*_*p*_*i*_ is convex (see also Tirole [5, pp. 221–223]). Dickson [47] showed that a concentration index can be expressed as in (16) with *w*_*i*_ (*i* = 1,…,*n*) being the firms’ so-called conjectural variation elasticities, with *w*_*i*_∈[0, 1] being the economically meaningful or valid interval for each *w*_*i*_.

Two of the members of (16) defined in Table 1 (*C*_7_ and *C*_17_) use weights *w*_*i*_ (*i* = 1,…,*n*) based on the ranks from the distributions *P*_*n*_ = (*p*_1_,…,*p*_*n*_) when ordered as in (1). The *C*_4_ is also based on ranks, but this index belongs to the reciprocal of the family in (16). With rank 1 for *p*_1_ (the largest *p*_*i*_), rank 2 for *p*_2_ (the second largest *p*_*i*_), etc., each of these three indices are decreasing functions of the weighted mean rank $\sum_{i=1}^{n}p_{i}i$.

Alternatively, instead of linearly decreasing weighted mean rank or the reciprocal weighted mean rank, consider the following weighted mean reciprocal rank:

$$C_{K}=\sum_{i=1}^{n}p_{i}/i$$ (17)

with the *p*_*i*_‘s ordered as in (1). This index is proposed as a new concentration index with desirable properties. In the case of market shares *p*_1_,…,*p*_*n*_, *C*_*K*_ is simply the market share of the largest firm (divided by 1), plus the market share of the second largest firm divided by 2,…., plus the market share of the *n*th largest (i.e., smallest) firm divided by *n*. The expression in (17) applies whether some or all of the *p*_*i*_‘s are equal. In the extreme case of $P_{n}^{0}$ in (4), $C_{K}(P_{n}^{0})=\sum_{i=1}^{n}\left(1/n)(1/i\right)$.

When there are ties among the *p*_*i*_‘s so that *p*_*i*_ = *p*_*i*+1_ = ⋯ = *p*_*i*+*k*_ = *p* for any *i* and *k*, it may appear as if different terms of *C*_*K*_ in (17) contribute differently to the value of *C*_*K*_ because their set of weights {1/*i*} differ even though the *p*_*i*_‘s are equal. However, the contribution toward *C*_*K*_ by such tied *p*_*i*_‘s can be expressed equivalently in terms of the arithmetic mean $\left(\sum_{j=i}^{i+k}1/j\right)/k$ of their reciprocal ranks as their common weight such that

$$p/i+p/(i+1)+\cdots +p/(i+k)=[\left(\sum_{j=i}^{i+k}1/j\right)/k](p+p+\cdots +p).$$

This expression shows clearly how the terms with equal *p*_*i*_‘s can all be considered as contributing equally toward the overall value of *C*_*K*_. Of course, $C_{K}(P_{n}^{0})$ represents the extreme case when *p*_1_ = ⋯ = *p*_*n*_ = 1/*n*.

**Fig 1:**
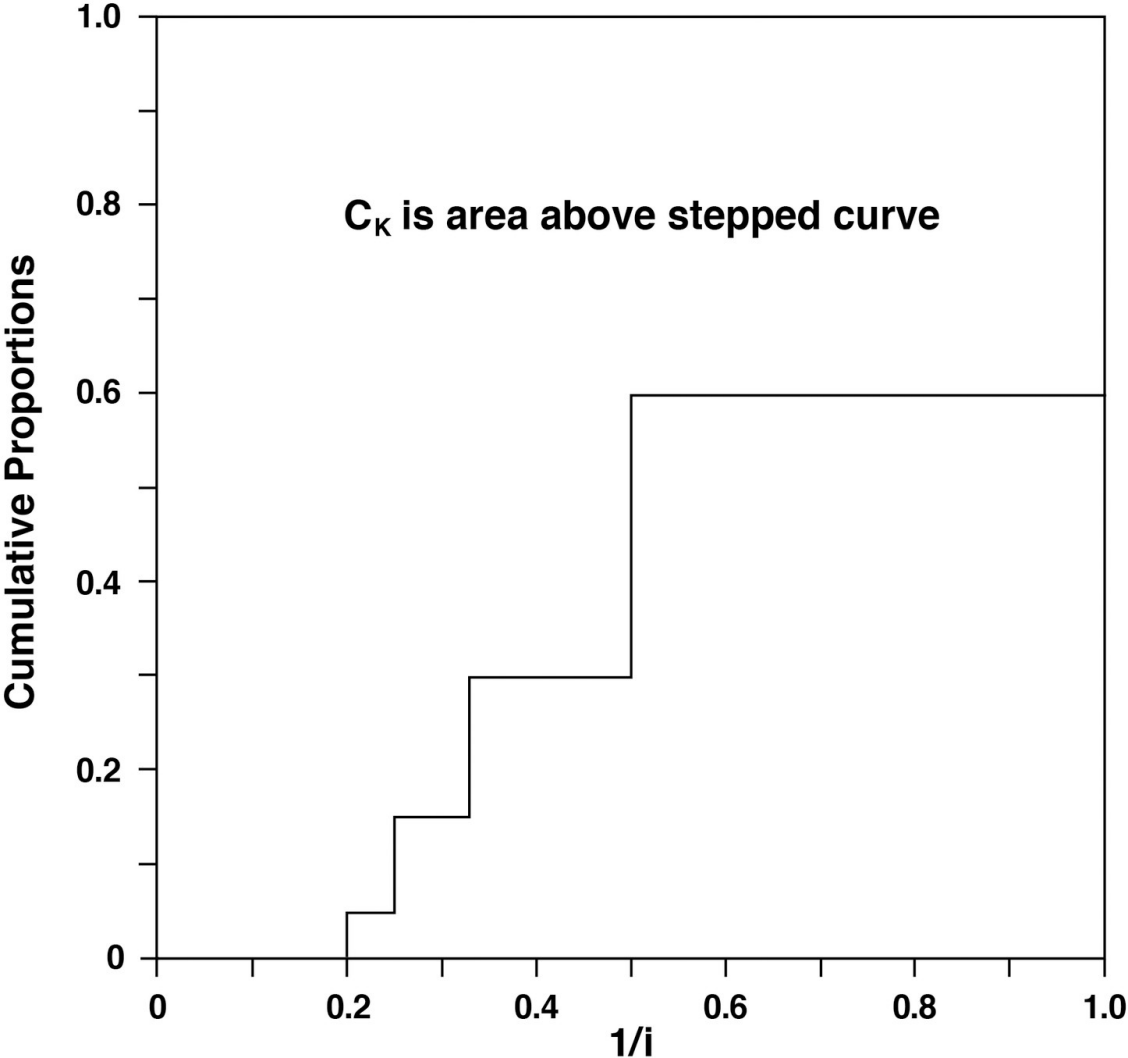
The value of *C*_*K*_ in (17) corresponds to the area above the step function formed by the cumulative *p*_*i*_‘s and the reciprocal rank 1/*i* where the cumulative proportions (*CP*) = 0 for 0<1/*i*<1/*n*; *CP* = *p*_*n*_ for 1/*n*≤1/*i*<1/(*n*−1); *CP* = *p*_*n*_+*p*_*n*−1_ for 1/(*n*−1)≤1/*i*<1/(*n*−2),…; *CP* = *p*_*n*_+*p*_*n*−1_+⋯+*p*_2_ for 1/2≤1/*i*<1; and *CP* = 1 for 1/*i* = 1. This exemplary graph is for the distribution *P*_5_ = (0.40, 0.30, 0.15, 0.10, 0.05) with *C*_*K*_ = 0.635.

Besides being a weighted mean of reciprocal ranks or a weighted sum of the *p*_*i*_‘s (ordered as in (1)), it may be of interest to note that *C*_*K*_ is also the reciprocal of the weighted harmonic mean of the ranks 1,2,…,*n*. The *C*_*K*_ can also be given a geometric interpretation in terms of the cumulative *p*_*i*_‘s and 1/*i* as explained and illustrated in Fig 1.

The range of values of *C*_*K*_ based on the extreme distributions in (4) is given by

$$C_{K}(P_{n}^{0})=(\frac{1}{n})\sum_{i=1}^{n}1/i\leq C_{K}(P_{n})\leq C_{K}(P_{n}^{1})=1.$$ (18)

The lower bound on *C*_*K*_ is simply the reciprocal of the harmonic mean of the first *n* positive integers (or the ranks 1,2,…,*n*). This is also by itself an interesting mathematical quantity. In particular, $H_{n}=\sum_{i=1}^{n}1/i$ is the partial sum of the harmonic series, also referred to as the *n-th harmonic number*. A recurrence relation for $C_{K}(P_{n}^{0})$ follows immediately from *H*_*n*+1_ = *H*_*n*_+1/(*n*+1). Although tabulated *H*_*n*_ values are readily available, a quick way to obtain values of $C_{K}(P_{n}^{0})$ is by means of the following formula:

$$C_{K}(P_{n}^{0})=[\log(n+1/2)+0.5772]/n$$ (19)

where the 0.5772 is the Euler-Mascheroni constant (to 4 decimal places). This formula is found to be correct to at least 3 decimal places for *n*>5.

### 4.2 Properties of *C*_*K*_

It is readily apparent from the expression in (17) that *C*_*K*_ has each of the Properties (P1)-(P3). The strict Schur-convexity of *C*_*K*_ (Property (P4)) is rather apparent from the form of (17) and the Schur-convexity definition in (2)–(3). It also follows from the fact that (a) *C*_*K*_ has the symmetry Property (P2) and the partial derivative ∂*C*_*K*_/∂*p*_*i*_ = 1/*i* is strictly decreasing in *i* = 1,…,*n* [32, p. 84].

What sets *C*_*K*_ apart from other concentration indices is the fact that *C*_*K*_ has the value-validity property (P5). From (10) and (17),

$$C_{K}(P_{n}^{\lambda })=\lambda +(1-\lambda )C_{K}(P_{n}^{0})$$ (20)

so that, with $C_{K}(P_{n}^{1})=1,\;C_{K}(P_{n}^{\lambda })$ complies with the value-validity condition in (11). The $C_{K}(P_{n}^{0})$ is the lower bound on *C*_*K*_ defined in (18).

Market concentration may be viewed as consisting of two different components: the size of a market or industry (*n*) and the inequality between the market shares. The index *C*_*K*_ can be formulated so as to reflect those two components separately by expressing *C*_*K*_(*P*_*n*_) as follows:

$$C_{K}(P_{n})=[C_{K}(P_{n}^{1})-C_{K}(P_{n}^{0})]C_{K}^{*}(P_{n})+C_{K}(P_{n}^{0}),\;C_{K}^{*}(P_{n})=\frac{C_{K}(P_{n})-C_{K}(P_{n}^{0})}{C_{K}(P_{n}^{1})-C_{K}(P_{n}^{0})}$$ (21)

with $C_{K}(P_{n}^{1})=1$ and $C_{K}^{*}(P_{n})\in [0,\;1]$. The $C_{K}(P_{n}^{0})$ is a function of *n* only and the normalized form $C_{K}^{*}(P_{n})$, which controls or adjusts for *n*, is a measure of market-share inequality. It is clear from (21) that for any fixed $C_{K}^{*}(P_{n})$, *C*_*K*_(*P*_*n*_) is a strictly increasing function of $C_{K}(P_{n}^{0})$ and strictly decreasing function of *n*. Similarly, for any fixed *n*, *C*_*K*_(*P*_*n*_) is strictly increasing in $C_{K}^{*}(P_{n})$. That is, *C*_*K*_(*P*_*n*_) increases as the size of the market decreases and as the inequality between the market shares increases.

The sensitivity of *C*_*K*_(*P*_*n*_) to changes in the inequality $C_{K}^{*}(P_{n})$ and to changes in *n* via $C_{K}(P_{n}^{0})$ can be compared by using partial derivatives and treating $C_{K}(P_{n}^{0})$ as a continuous variable for mathematical purpose. Then, it follows from (21) that

$$\frac{\partial C_{K}(P_{n})}{\partial C_{K}^{*}(P_{n})}=(\frac{1-C_{K}(P_{n}^{0})}{1-C_{K}^{*}(P_{n})})\frac{\partial C_{K}(P_{n})}{\partial C_{K}(P_{n}^{0})}$$ (22)

It appears from (22) that whether *C*_*K*_(*P*_*n*_) is more sensitive to changes in $C_{K}^{*}(P_{n})$ than to changes in $C_{K}(P_{n}^{0})$ or vice versa depends on whether $C_{K}^{*}(P_{n})>C_{K}(P_{n}^{0})$ or $C_{K}^{*}(P_{n})<C_{K}(P_{n}^{0})$.

As an extension of the weighted mean of the two extremal distributions $P_{n}^{0}$ and $P_{n}^{1}$ in (10), consider the weighted mean of any two distributions *P*_*n*_ = (*p*_1_,…,*p*_*n*_) and *Q*_*n*_ = (*q*_1_,…,*q*_*n*_), both arranged in descending order as in (1). It then follows immediately from the definition of *C*_*K*_ in (17) that the value of *C*_*K*_ for the mixture distribution with weights *w* and 1-*w* is given by

$$C_{K}[wP_{n}+(1-w)Q_{n}]=wC_{K}(P_{n})+(1-w)C_{K}(Q_{n}),\;0\leq w\leq 1.$$ (23)

This property of *C*_*K*_, which would seem to be an intuitively reasonable one, is basically an extension of the value-validity requirement in (11). Thus, if an index complies with (23), it will necessarily comply with (11) since $P_{n}^{0}$ and $P_{n}^{1}$ are simply special forms of *Q*_*n*_ and *P*_*n*_, respectively.

The *C*_*K*_ does not have the replication property, although, as discussed in Section 2.1, this property cannot be considered essential and may rather be a questionable one. In fact, the replication property and the value-validity property (P5) are inconsistent or mutually exclusive. This inconsistency can be proved by first noting that for the distribution $P_{n}^{\lambda }$ in (9) and analogously to (21), the value-validity condition in (11) can also be expressed as

$$C^{*}(P_{n}^{\lambda })=\frac{C(P_{n}^{\lambda })-C(P_{n}^{0})}{C(P_{n}^{1})-C(P_{n}^{0})}=\lambda =(\frac{n}{\sqrt{n-1}})s_{n}=s_{n}^{*}(P_{n}^{\lambda })$$ (24)

where *s*_*n*_ is the standard deviation (with devisor *n*) of the *n* components of $P_{n}^{\lambda }$ and $s_{n}^{*}(P_{n}^{\lambda })$ is its normalized form. If *C* were to have the replication property, then $C(P_{n}^{\lambda }/k)=C\left(P_{n}^{\lambda }\right)/k,\;C(P_{n}^{0}/k)=C\left(P_{n}^{o}\right)/k$, and $C(P_{n}^{1}/k)=C\left(P_{n}^{1}\right)/k$ so that $C^{*}(P_{n}^{\lambda }/k)=C^{*}(P_{n}^{\lambda })$. However, since $s_{kn}(P_{n}^{\lambda }/k)=s_{n}\left(P_{n}^{\lambda }\right)/k$ and hence $s_{kn}^{*}(P_{n}^{\lambda }/k)\neq s_{n}^{*}(P_{n}^{\lambda })$, *C* cannot simultaneously have the replication property and meet the condition in (24) for value validity.

Some prefer to use a concentration index that has the property of being a so-called *numbers equivalent* index such as the index family *C*_*11*_ by Hannah and Kay [3] defined in Table 1. The numbers equivalent *NC*_*K*_ of *C*_*K*_ in (17) can be obtained by simply setting $C_{K}(P_{n})=C_{K}(P_{NC_{K}}^{0})=\left(\sum_{i=1}^{NC_{K}}1/i\right)/NC_{K}$ and solving for the nearest integer *NC*_*K*_ for any given *P*_*n*_. Thus, for the concentration *C*_*K*_(*P*_*n*_) of the market-share distribution *P*_*n*_ = (*p*_1_,…,*p*_*n*_), *NC*_*K*_ becomes the number of firms in an equivalent market with equal market shares and whose market concentration equals the given *C*_*K*_(*P*_*n*_). However, while *NC*_*K*_ does provide an alternative interpretation for any index value *C*_*K*_(*P*_*n*_), its utility is limited by its lack of the value-validity property (P5) since *NC*_*K*_ is not a linear function of *C*_*K*_(*P*_*n*_) as would be required for compliance with (11).

Another aid for interpreting or visualizing values of *C*_*K*_ may be the use of the *equivalent lambda distribution* $P_{n}^{\lambda_{e}}$ obtained from (10) and (14) and by setting $C_{K}(P_{n})=C_{K}(P_{n}^{\lambda })$ for the $C_{K}(P_{n}^{\lambda })$ in (20) and solving for *λ* = *λ*_*e*_ and for any distribution *P*_*n*_ = (*p*_1_,…,*p*_*n*_). This solution is seen to equal the normalized $C_{K}^{*}(P_{n})$ in (21), i.e., $\lambda_{e}=C_{K}^{*}(P_{n}).$ Thus, for example, consider *P*_*5*_ = (0.40, 0.30, 0.15, 0.10, 0.05) for which *C*_*K*_(*P*_5_) = 0.64 and $C_{K}(P_{{}_{5}}^{0})=0.46$ so that $\lambda_{e}=C_{{}_{K}}^{*}(P_{5})=0.33$. This gives $P_{5}^{0.33}=(0.48,\;0.13,\ldots ,\;0.13)$ as being the equivalent lambda distribution having (approximately) the same *C*_*K*_-value as *C*_*K*_(*P*_5_) for the given *P*_*5*_.

## 5. Comparison with other indices

The most significant difference between the new index *C*_*K*_ in (17) and other indices proposed to date is due to the fact that *C*_*K*_ has the value-validity property (P5) whereas most other indices lack this property. However, the most interesting comparison is between *C*_*K*_ and the two indices *CR*_*m*_ and *HHI* since those are by far the most popular ones (e.g., [13, pp.1216-118], [14, pp. 97–101], [6, Ch. 8]). Among the *CR*_*m*_ members, the most commonly used member is the four-firm concentration ratio (*CR*_4_) (e.g., [16, p. 255]). Comparisons between *HHI* and *CR*_4_ have been extensively discussed [15].

In order to compare the values of *C*_*K*_ with those of *HHI* and *CR*_4_ for a wide variety of market-share distributions *P*_*n*_ = (*p*_1_,…,*p*_*n*_), a computer algorithm was used to randomly generate *P*_*n*_ as follows. First, *n* was generated as a random integer between *n* = 5 and *n* = 100 (inclusive). The lower limit *n* = 5 was chosen since the four-firm *CR*_4_ was used and to avoid *CR*_4_ = 1 values simply because of values *n<*5 being generated. Then, for each such generated *n*, each *p*_*i*_ (ordered as in (1)) was generated as a random number (to the desired decimals) within the following intervals:

$$\frac{1}{n}\leq p_{1}\leq 1$$

$$\frac{1-\sum_{j=1}^{i-1}p_{j}}{n-(i-1)}\leq p_{i}\leq \min\{p_{i-1},\;1-\sum_{j=1}^{i-1}p_{j}\}\;\mathrm{for}\;i=2,\ldots ,n-1$$

$$p_{n}=1-\sum_{j=1}^{n-1}p_{j}.$$

**Fig 2:**
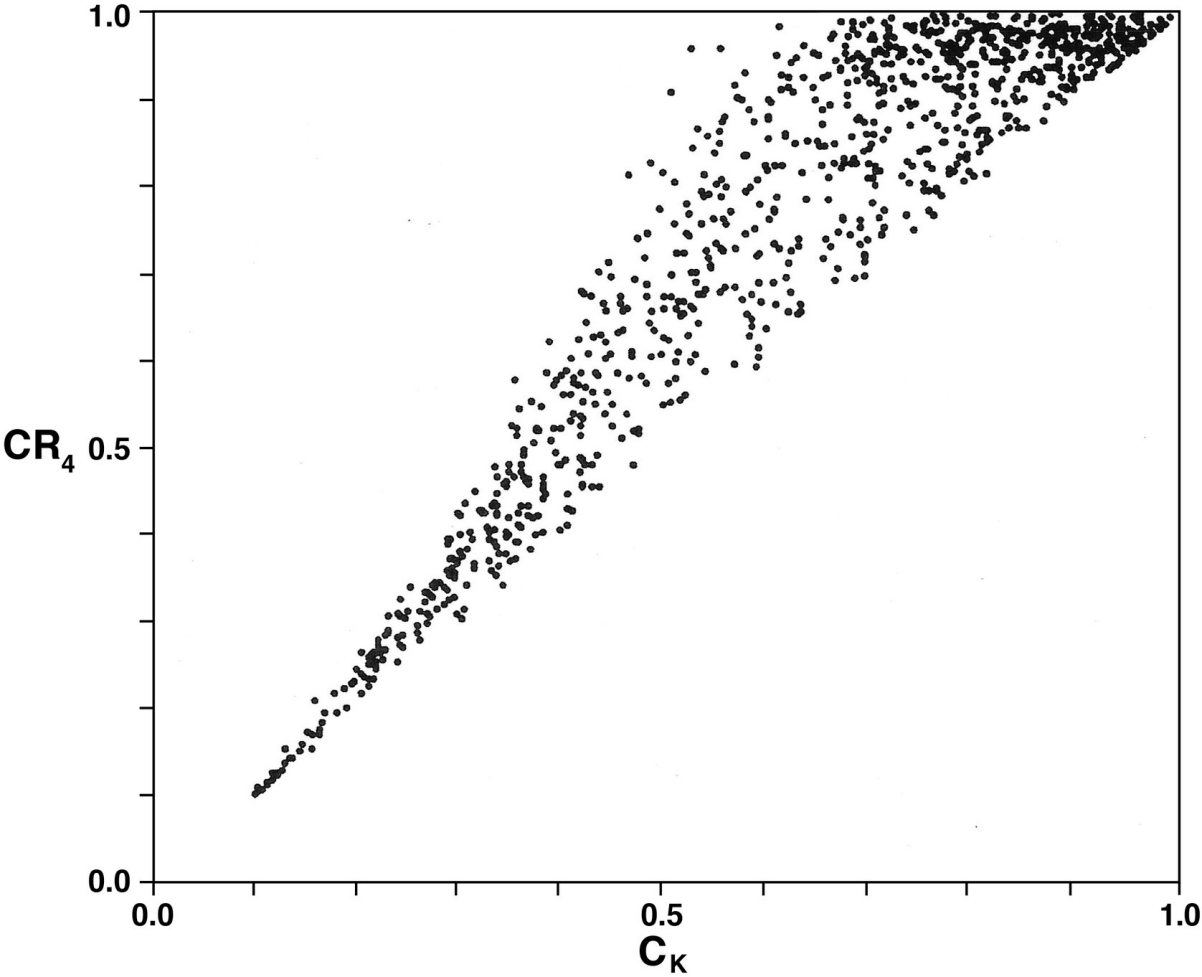
Comparison of values of *CR*_4_ and *C*_*K*_ for 1000 randomly generated market-share distributions *P*_*n*_ = (*p*_1_,…,*p*_*n*_) with the number of firms *n* varying as a random integer between 5 and 100.

A total of 1000 such distributions were generated and the corresponding values of *C*_*K*_, *CR*_4_, and *HHI* were computed. The results for the *C*_*K*_−*CR*_4_ comparison is illustrated in Fig 2.

It is clear from their differing expressions that there cannot be any precise functional relationship between *CR*_4_ and *C*_*K*_ as is also demonstrated in the result in Fig 2. The rather systematic result is that the variation in *CR*_4_-values for any *C*_*K*_-value tends to increase quite dramatically with increasing *C*_*K*_. More specifically, the change in the variation of *CR*_4_-values tends to increase up to about *C*_*K*_ = 0.5 beyond which it decreases. The same type of general relationship is also found between *CR*_4_ and *HHI* [15].

**Fig 3:**
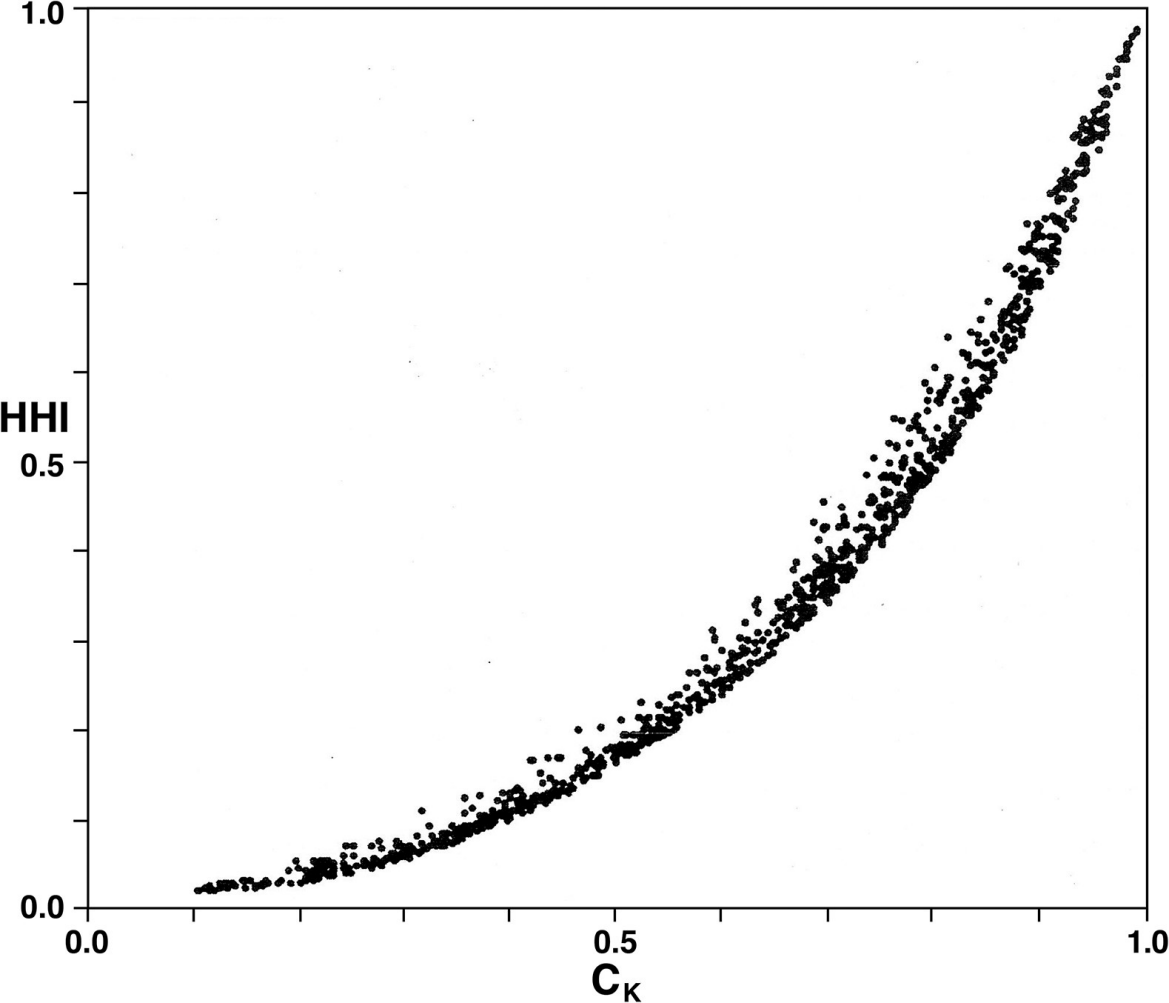
Comparison of values of *HHI* and *C*_*K*_ for 1000 randomly generated market-share distributions *P*_*n*_ = (*p*_1_,…,*p*_*n*_) with the number of firms *n* varying as a random integer between 5 and 100.

From the simulation results for *HHI* versus *C*_*K*_ in Fig 3, it is rather strikingly clear that for any given value of either *C*_*K*_ or *HHI*, the variation in the other index is quite limited. In fact, this scatter diagram supports the proposition that a functional relationship exists between the two indices.

In order to explore this potential relationship, three additional sets of distributions *P*_*n*_ have been analyzed: (1) the lambda distribution in (10), (2) randomly generated *P*_*n*_ as described above, and (3) real market-share distributions. The real data consist of the market shares of the firms within 20 different markets and were chosen so as to be readily available, include a wide variety of markets, and cover a reasonably wide range of concentration values. Those 20 data sets were simply obtained from internet searches with the source of each being identified. The purpose of using those real data sets was for exploring the potential relationship between *C*_*K*_ and *HHI* rather than providing realiable and representative market concentration results for specific markets. The same data sources were also used in Kvålseth [15] for comparing *CR*_4_ and *HHI*.

Those three different types of market-share data were then used to explore potential functions and transformations that would provide a reasonably accurate description of the apparent relationship between *C*_*K*_ and *HHI*. The following result has emerged from this analysis:

$$C_{K}\approx C_{KH}=(\frac{1}{3})\log[(e^{3}-1)HHI+1]$$ (25)

for any distribution *P*_*n*_ = (*p*_1_,…,*p*_*n*_) where the logarithm is to base *e* (natural logarithm) and the exponential term *e*^3^−1 = 19.0855. The approximation between *C*_*K*_ and the transformation *C*_*KH*_ in (25) results from regression analysis. Specifically, for the regression (through the origin) model *C*_*K*_ = *αC*_*KH*_, the following estimates are obtained: $\hat{\alpha }=0.98$ for the data in Table 2 involving $P_{n}^{\lambda }$ in (10) and different values of *n* and *λ*, $\hat{\alpha }=1.00$ for the data in Table 3 involving randomly generated *P*_*n*_, and $\hat{\alpha }=1.02$ for the real market results in Table 4.

**Table 2:** Values of *C*_*K*_ in (17), *HHI* defined in Table 1, and *C*_*KH*_ in (25) for $P_{n}^{\lambda }$ in (9) with varying *λ* and *n*.

*λ*	*n*	*C*_*K*_	*HHI*	*C*_*KH*_
0.10	2	0.78	0.51	0.79
0.30	2	0.83	0.55	0.81
0.50	2	0.88	0.63	0.86
0.70	2	0.93	0.75	0.91
0.90	2	0.98	0.91	0.97
0.10	5	0.51	0.21	0.54
0.30	5	0.62	0.27	0.61
0.50	5	0.73	0.40	0.72
0.70	5	0.84	0.59	0.84
0.90	5	0.95	0.85	0.95
0.10	10	0.36	0.11	0.38
0.30	10	0.51	0.18	0.50
0.50	10	0.65	0.33	0.66
0.70	10	0.79	0.54	0.81
0.90	10	0.93	0.83	0.94
0.10	20	0.26	0.06	0.25
0.30	20	0.43	0.14	0.43
0.50	20	0.59	0.29	0.63
0.70	20	0.75	0.52	0.80
0.90	20	0.92	0.82	0.94
0.10	30	0.22	0.04	0.19
0.30	30	0.39	0.12	0.40
0.50	30	0.57	0.28	0.62
0.70	30	0.74	0.51	0.79
0.90	30	0.91	0.82	0.94
0.10	50	0.18	0.03	0.15
0.30	50	0.36	0.11	0.38
0.50	50	0.54	0.27	0.61
0.70	50	0.73	0.50	0.79
0.90	50	0.91	0.81	0.93

**Table 3:** Values of *C*_*K*_ in (17), $C_{K}^{*}$ in (21) and (26), d* in (26), *HHI* and *CR*_4_ defined in Table 1, and *C*_*KH*_ in (25) for randomly generated *P*_*n*_ = (*p*_1_,…,*p*_*n*_) and 2≤*n*≤30.

n	*C*_*K*_	$C_{K}^{*}$	d*	*HHI*	*CR*_4_	*C*_*KH*_
27	0.84	0.81	0.76	0.59	0.94	0.84
29	0.16	0.03	0.03	0.04	0.18	0.17
19	0.75	0.69	0.68	0.49	0.79	0.78
8	0.50	0.24	0.23	0.17	0.63	0.49
12	0.45	0.26	0.24	0.14	0.55	0.43
9	0.71	0.58	0.53	0.36	0.96	0.69
15	0.48	0.33	0.32	0.16	0.54	0.47
3	0.79	0.46	0.47	0.48	1.00	0.77
14	0.24	0.01	0.01	0.07	0.30	0.29
4	0.72	0.42	0.40	0.37	1.00	0.70
21	0.35	0.22	0.20	0.09	0.38	0.33
24	0.36	0.24	0.21	0.08	0.42	0.32
13	0.82	0.76	0.72	0.56	0.89	0.82
12	0.91	0.88	0.85	0.75	0.95	0.91
15	0.24	0.02	0.02	0.07	0.29	0.28
29	0.60	0.54	0.48	0.26	0.78	0.60
20	0.26	0.10	0.09	0.06	0.31	0.25
8	0.77	0.65	0.60	0.44	1.00	0.75
13	0.40	0.21	0.19	0.11	0.53	0.37
17	0.92	0.90	0.88	0.78	0.95	0.92
19	0.38	0.24	0.22	0.10	0.43	0.36
14	0.28	0.06	0.05	0.07	0.36	0.29
28	0.29	0.17	0.16	0.06	0.35	0.25
17	0.65	0.56	0.52	0.32	0.73	0.65
25	0.49	0.40	0.39	0.19	0.50	0.50
17	0.91	0.89	0.85	0.74	0.97	0.90
25	0.36	0.24	0.22	0.08	0.44	0.32
21	0.18	0.01	0.01	0.05	0.20	0.22
29	0.34	0.24	0.22	0.08	0.37	0.31
23	0.78	0.74	0.68	0.49	0.98	0.78

**Table 4:** Values of *C*_*K*_ in (17), $C_{K}^{*}$ in (21) and (26), d* in (26), *HHI* defined in Table 1, and *C*_*KH*_ in (25) for a sample of real market-share data.

n	*C*_*K*_	$C_{K}^{*}$	d*	*HHI*	*CR*_4_	*C*_*KH*_	Source	(Market type)
16	0.37	0.20	0.20	0.10	0.50	0.36	[48]	(Airline travel)
16	0.42	0.27	0.25	0.12	0.60	0.40	[48]	(Airline travel)
8	0.47	0.20	0.20	0.16	0.75	0.47	[49]	(U.S. distilled liquor)
10	0.45	0.22	0.21	0.14	0.64	0.43	[50]	(Paints, coatings)
10	0.38	0.12	0.11	0.11	0.52	0.38	[51]	(Pharmaceuticals)
15	0.39	0.22	0.19	0.10	0.54	0.36	[52]	(Insurances companies)
12	0.52	0.35	0.32	0.18	0.69	0.50	[53]	(Weapons exporters)
30	0.26	0.15	0.13	0.05	0.34	0.22	[54]	(Car sales, Britain)
12	0.39	0.18	0.20	0.12	0.60	0.40	[55]	(Auto Mnf., US)
8	0.49	0.23	0.25	0.18	0.77	0.50	[50]	(Craft beer, US)
9	0.46	0.21	0.23	0.16	0.75	0.47	[50]	(Running shoe sales)
5	0.97	0.94	0.94	0.91	1.00	0.97	[56]	(Search eng., Norway)
4	0.71	0.39	0.38	0.36	1.00	0.69	[57]	(Comm. water heaters)
3	0.90	0.74	0.74	0.70	1.00	0.89	[58]	(Microprocessors)
10	0.51	0.31	0.28	0.17	0.72	0.48	[50]	(Top charter airlines)
5	0.58	0.23	0.22	0.24	0.79	0.57	[59]	(Global cigarettes, 2019)
10	0.52	0.32	0.30	0.18	0.68	0.50	[50]	(Farm mach., equip.)
20	0.32	0.17	0.18	0.08	0.48	0.31	[60]	(Global car sales)
10	0.40	0.15	0.15	0.12	0.60	0.40	[50]	(Top airlines, world)
8	0.70	0.55	0.51	0.35	0.83	0.68	[61]	(Consumer products)

In fact, for $\hat{\alpha }=1.00$, the fitted model ${\hat{C}}_{K}=C_{KH}$ provides an excellent fit to all data sets. The coefficient of determination, when properly computed [62], is found to be $R^{2}=1-{\sum {\left(C_{K}-C_{KH}\right)}^{2}/\sum \left(C_{K}-{\bar{C}}_{K}\right)}^{2}=0.985,\;0.990,\;\mathrm{and}\;0.990$ for the data in Tables 2–4, respectively. That is, 99% of the variation of *C*_*K*_ (about its mean) is explained (accounted for) by the fitted model.

**Fig 4:**
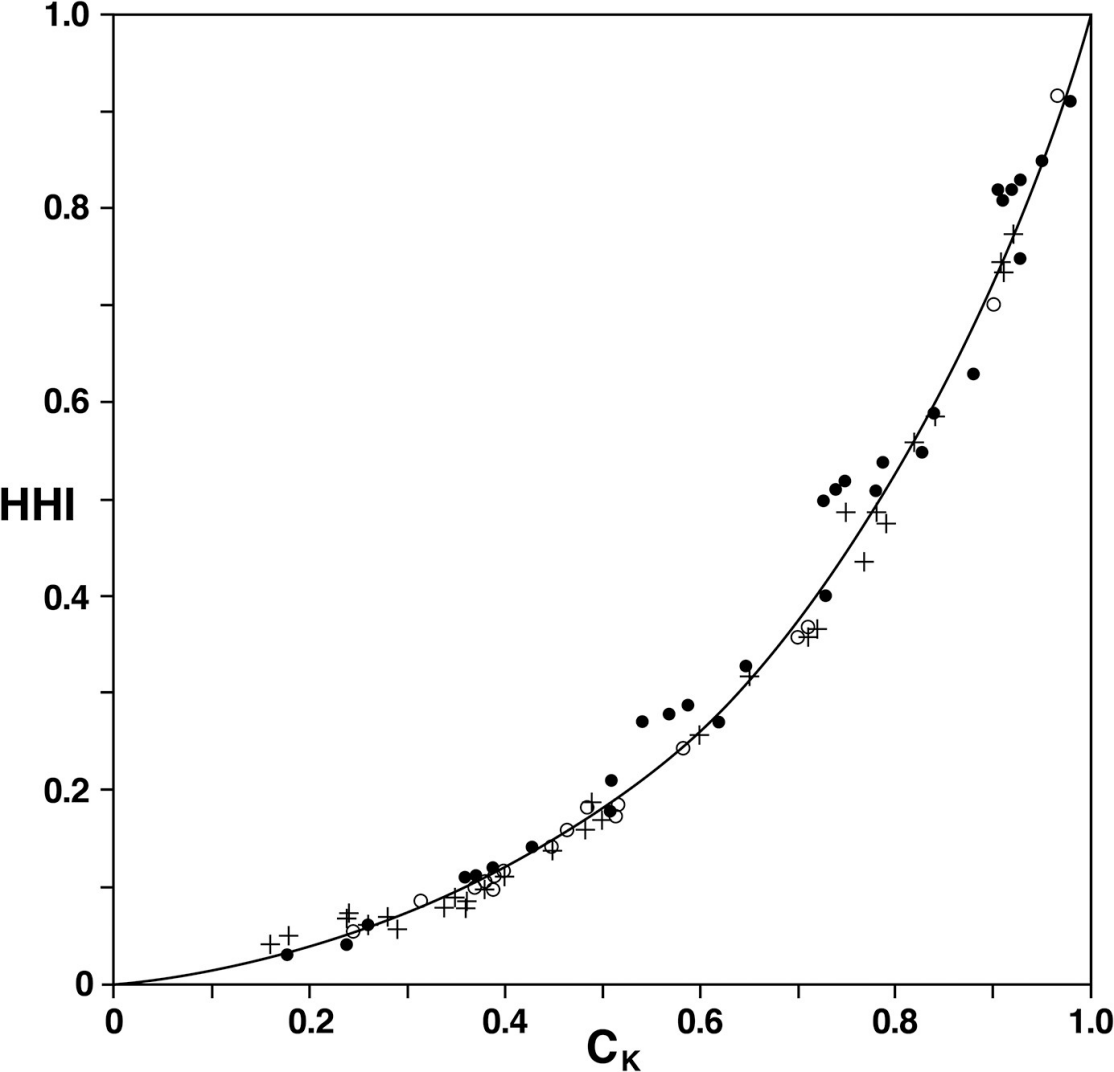
Scatter diagram of *HHI* versus *C*_*K*_ from the data in Table 2 (dots), Table 3 (crosses) and Table 4 (circles). The curve represents the fitted model in (25).

While the excellent fit of the relationship *C*_*K*_≈*C*_*KH*_ in (25) is apparent from the data in Tables 2–4, it is more conveniently depicted in Fig 4. It is clear from this scatter diagram that (25) is indeed an appropriate relationship supported by the data. When looking at the residuals (*C*_*K*_−*C*_*KH*_) in Fig 4, they tend to fall within a rather uniform band along each side of the curve. There appears to be no other systematic pattern among the residuals that would indicate the need for any alternative expression.

Tables **3** and **4** also give the results for the following normalized forms of *C*_*K*_ and of the Euclidean distance $d(P_{n},P_{n}^{0})$ (generalized from (8) and (13)):

$$C_{K}^{*}(P_{n})=\frac{C_{K}(P_{n})-C_{K}(P_{n}^{0})}{1-C_{K}(P_{n}^{0})},\;d^{*}(P_{n},P_{n}^{0})=\frac{d(P_{n},P_{n}^{0})}{d(P_{n}^{1},P_{n}^{0})}.$$ (26)

It is clear from these results that the approximation $C_{K}^{*}(P_{n})\approx d^{*}(P_{n},P_{n}^{0})$ holds to a highly respectable degree of accuracy. In fact, the properly computed *R*^*2*^*-*values for the fitted model ${\hat{C}}_{K}^{*}(P_{n})=d^{*}(P_{n},P_{n}^{0})$ are found to be $R^{2}=1-{\sum {\left(C_{K}^{*}-d^{*}\right)}^{2}/\sum \left(C_{K}^{*}-{\bar{C}}_{K}^{*}\right)}^{2}=0.990$ for all the data in each of Tables **3** and **4**.

In many practical situations involving potential concerns about mergers and market structure, the number of firms *n* within a market or industry may be quite small. Although the data in Tables 2–4 include a number of data sets with *n*≤10, it may be worthwhile to look at some additional data as in Table 5 obtained for market-share distributions *P*_*n*_ = (*p*_1_,…,*p*_*n*_) computer generated from the algorithm described above for 2≤*n*≤10.

**Table 5:** Values of *C*_*K*_ in (17), $C_{K}^{*}$in (21), d* in (26), *HHI* defined in Table 1, and *C*_*KH*_ in (25) for randomly generated *P*_*n*_ = (*p*_1_,…,*p*_*n*_) and 2≤*n*≤10.

n	*C*_*K*_	$C_{K}^{*}$	d*	*HHI*	*C*_*KH*_
2	0.99	0.96	0.94	0.94	0.98
9	0.79	0.70	0.65	0.49	0.78
4	0.87	0.73	0.72	0.64	0.86
5	0.57	0.21	0.22	0.24	0.57
6	0.65	0.41	0.40	0.30	0.64
5	0.68	0.41	0.43	0.35	0.68
7	0.61	0.38	0.37	0.26	0.60
2	0.76	0.04	0.00	0.50	0.79
5	0.56	0.19	0.22	0.24	0.57
4	0.67	0.31	0.33	0.33	0.66
6	0.92	0.86	0.83	0.74	0.91
8	0.70	0.55	0.56	0.40	0.72
8	0.50	0.24	0.23	0.17	0.48
3	0.71	0.26	0.29	0.39	0.71
5	0.63	0.32	0.35	0.30	0.64
9	0.96	0.94	0.94	0.90	0.97
5	0.46	0.01	0.00	0.20	0.52
8	0.59	0.38	0.36	0.24	0.57
5	0.64	0.34	0.32	0.28	0.62
8	0.65	0.47	0.42	0.28	0.62
3	0.82	0.54	0.51	0.51	0.79
7	0.70	0.52	0.51	0.37	0.70
10	0.61	0.45	0.45	0.28	0.62
8	0.97	0.95	0.95	0.92	0.97
10	0.58	0.41	0.38	0.23	0.56
6	0.57	0.27	0.28	0.23	0.56
10	0.93	0.90	0.86	0.77	0.92
7	0.43	0.10	0.09	0.15	0.45
3	0.69	0.21	0.23	0.37	0.70
4	0.97	0.94	0.93	0.90	0.97

These results clearly support those in Table 2 for 2≤*n*≤50, Table 3 for 3≤*n*≤29, and Table 4 for 3≤*n*≤30. Again, the relationship between *C*_*K*_ and *HHI* is quite accurately described by (25), with *R*^2^ = 0.986 for the fitted model ${\hat{C}}_{K}=C_{KH}$ and the data in Table 5. Similarly, the normalized $C_{K}^{*}\in [0,\;1]$ and the normalized Euclidean distance d*∈[0, 1] show an impressive agreement, with *R*^2^ = 0.993 for the fitted model ${\hat{C}}_{K}^{*}=d^{*}$ and the data in Table 5.

This close approximation between $C_{K}^{*}(P_{n})$ and $d^{*}(P_{n},P_{n}^{0})$ has important implications. First, it provides $C_{K}^{*}(P_{n})$ with an interesting mathematical property: $C_{K}^{*}(P_{n})$ becomes approximately a normalized distance metric (see, e.g., Chen et al. [63] for such metrics). This is of significance since the metric property is a strong one with far-reaching mathematical consequences. Second, this close approximation between $C_{K}^{*}(P_{n})$ and $d^{*}(P_{n},P_{n}^{0})$ also means that *C*_*K*_(*P*_*n*_) is an approximately linear function of the standard deviation *s*_*n*_ of *p*_1_,…,*p*_*n*_ (with devisor *n*). Specifically, it readily follows that

$$C_{K}(P_{n})\approx [1-C_{K}(P_{n}^{0})](\frac{n}{\sqrt{n-1}})s_{n}+C_{K}(P_{n}^{0}).$$ (27)

In the case of $P_{n}=P_{n}^{\lambda }$ in (10) and since *C*_*K*_ satisfies (11)–(13), the relationship in (27) becomes an equality as is also implied by (24). Therefore, because of the equality in (14) and from (24), the approximate relationship in (27) is not an unexpected one. It is important because it shows that, similarly to (21), *C*_*K*_(*P*_*n*_) is a function of both the number of firms in a market and their market-share variation as measured by the well-known standard deviation.

For the sake of completeness, the results for *CR*_4_ are also given in Tables 3 and 4. Even though there is a substantial correlation between *C*_*K*_ and *CR*_4_ (Pearson’s *r* = 0.97 and 0.92 and Spearman’s *r*_*s*_ = 0.89 and 0.95 for Tables 3 and 4, respectively), there are also substantial individual differences. Such differences between the values of *C*_*K*_ and *CR*_4_ for individual data points seem to increase with increasing values of the two indices as in Fig 2. The *HHI* and *CR*_4_ are also highly correlated with *r* = 0.89 and *r*_*s*_ = 0.91 and *r* = 0.81 and *r*_*s*_ = 0.96 for Tables 3 and 4, respectively. In spite of such high correlations, the different indices can certainly produce very conflicting results.

## 6. Discussion

### 6.1 Comments on (25)

The approximate, although quite accurate, relationship between *C*_*K*_ and *HHI* in (25) has important implications. First, with *HHI* becoming the increasingly dominant concentration index [7], reported *HHI* values can be converted into the corresponding *C*_*K*_ values for valid comparisons and interpretations of market concentrations.

Second, while the varying sensitivity of *HHI* to small changes in market-share distributions was discussed above based on (15) and on the particular distribution $P_{n}^{\lambda }$ in (10), this analysis can be done for any distribution *P*_*n*_ by using (25). With *C*_*K*_ and *HHI* being functions of *P*_*n*_ and taking the derivatives of both sides of (25) with respect to *P*_*n*_ gives the following approximate relationship:

$$\frac{dHHI(P_{n})}{dP_{n}}\approx A\frac{dC_{K}(P_{n})}{dP_{n}},\;A=3[HHI(P_{n})+{\left(e^{3}-1\right)}^{-1}].$$ (28)

Compared with *C*_*K*_, which has constant sensitivity or discriminant power because of its value-validity property (P5), that of *HHI* is seen from (28) to increase with increasing *HHI*-values. Since *A* = 1 for *HHI* = 0.32 in (28) and since *C*_*K*_ behaves appropriately, the implication is that *HHI* lacks adequate sensitivity when *HHI*<0.32 and has excessive and rapidly increasing sensitivity for *HHI*>0.32. This result is not inconsistent with the more general representation by the data in Fig 4.

### 6.2 *C*_*K*_ and economic theory

One of the major reasons for the popularity of *HHI* is its solid theoretical relationship with market power. Cowling and Waterson [64], for example, showed that for an oligopolistic market (industry) with profit-maximizing firms, the average price-cost margin is directly related to the *HHI* index. See also Martin [65, pp. 150–151, 337–338] and Carlton and Perloff [16, p. 283].

More specifically, the price-cost margin of Firm *i* is defined as *PCM*_*i*_ = (*P*_*i*_−*MC*_*i*_)/*P*_*i*_ where *P*_*i*_ is the market price set by the firm and *MC*_*i*_ is the firm’s marginal cost. The *PCM*_*i*_, which is also the well-known Lerner index, is considered as a measure of a firm’s market power. Then, as shown by Cowling and Waterson [64] and under certain economic assumptions (including the assumption that *P*_*i*_ = *P* for all *i*), the weighted mean (*PCM*) of all the *PCM*_*i*_‘s within a market or an industry, using the market shares as the weights $\left(i.e.,\;PCM=\sum_{i=1}^{n}p_{i}PCM_{i}\right)$, is related to *HHI* as follows:

PCM=HHI/|η| (29)

where *η* is the market (industry) price elasticity of demand. The price elasticity of demand, which measures the variation in demand in response to a variation in price, is generally negative so that the right side in (29) may also be expressed as −*HHI*/*η*.

From (29), and by inverting the expression in (25), it follows that

$$PCM\approx (\frac{1}{\left|\eta \right|})(\frac{e^{3C_{K}}-1}{e^{3}-1}).$$ (30)

That is, while the relationship between *PCM* and *HHI* is linear, the *PCM* is related to *C*_*K*_ in terms of an approximate exponential function. Since the close approximation in (25) has been empirically established, the approximation in (30) can be expected to be similarly close. Consequently, with *PCM* being a well-known measure of market power, the relation in (30) shows that increasing market concentration as measured by *C*_*K*_ results in increasing market power and hence decreasing competition and efficiency.

### 6.3 *C*_*K*_ and merger implications

The *HHI* is often computed from market shares as being percentages (rather than probabilities or proportions), which is the most typical form for reporting such data. Thus, the potential values of *HHI* range from 0 to 10,000 as is also used in the DOJ and FTC Merger Guidelines. For any *HHI*∈(0, 10,000], the corresponding values of *C*_*K*_ in percentage points can be obtained from (25) as

$$C_{K}\approx C_{KH}=(\frac{100}{3})\log[(e^{3}-1)(\frac{HHI}{10,000})+1]\in \left(0,\;100\right].$$ (31)

Based on (31), the most recent (2010) Horizontal Merger Guidelines may then be summarized in terms of *C*_*K*_*-*values and changes Δ*C*_*K*_ as follows:

- *Small Change in Concentration*: Mergers involving an increase Δ*C*_*K*_<2 percentage points are unlikely to have adverse competitive effects and ordinarily require no further analysis.
- *Unconcentrated Markets* (*C*_*K*_<45): Mergers resulting in unconcentrated markets are unlikely to have adverse competitive effects and ordinarily require no further analysis.
- *Moderately Concentrated Markets* (45<*C*_*K*_<58): Mergers resulting in moderately concentrated markets that involve Δ*C*_*K*_>2 potentially raise significant competitive concerns and often warrant scrutiny.
- *Highly Concentrated Markets* (*C*_*K*_>58): Mergers resulting in highly concentrated markets that involve 2<Δ*C*_*K*_<3 potentially raise significant competitive concerns and often warrant scrutiny. Mergers resulting in highly concentrated markets that involve Δ*C*_*K*_>3 will be assumed to likely enhance market power. The presumption may be rebutted by persuasive evidence showing that the merger is unlikely to enhance market power.

Similarly, the European Commission (EC) Merger Guidelines (Regulation) also uses the *HHI* index [13, p. 120] [66]. The EC guidelines may be expressed in terms of *C*_*K*_ in percentage points as follows:

- It is unlikely that a merger will cause horizontal competition concerns if (a) the post-merger *C*_*K*_<36, (b) the post-merger *C*_*K*_ is between 36 and 52 and Δ*C*_*K*_<4,or (c) the post-merger *C*_*K*_>52 and Δ*C*_*K*_<2, except in case of special given circumstances.

While these guidelines are directly transferred from those using *HHI*, it is important to note that since *HHI* lacks the value-validity property (P5), changes Δ*HHI* in *HHI*-values do not truly reflect changes in the extent of the concentration characteristic, but rather reflect changes in *HHI* values. As discussed above, the sensitivity of *HHI* to small changes in the market-share distribution *P*_*n*_, rather than being constant as in the case of *C*_*K*_, increases substantially with increasing *HHI* values. Thus, for example, an increase of Δ*HHI* = 100 at *HHI* = 1,500 and at *HHI* = 2,500, as used by the Merger Guidelines, cannot be considered as equivalent changes in concentration. In fact, the corresponding changes in *C*_*K*_ from (25), rather than being equal, are found to be 1.7 and 1.1, respectively.

Such invalid assumptions or interpretations of changes in *HHI* are of increasing importance, both theoretically and empirically, from studies emphasizing changes in concentration over its actual level. For example, Nocke and Whinston [8] show “that there is both a theoretical and empirical basis for focusing solely on the change in the Herfindahl index, and ignoring its level, in screening mergers for whether their unilateral effect will harm consumers (p.1).” Similarly, Miller et al. [67] used a Monte Carlo simulation study to evaluate price effects of mergers, finding a strong correlation between Δ*HHI* and the price change.

Whatever the purpose is of an investigation involving market concentration, whether theoretical or empirical, one proposition seems clear: changes in the values of a concentration index may be as important, if not more so, than its individual index values. Results that truly represent reality require an index with the value-validity property (P5). The *C*_*K*_ is claimed to be such an index.

## 7. Conclusion

Value validity is introduced as a property required of a concentration index in order for its numerical values and their changes to provide reliable and true representations of the market or industry concentration characteristic. Since other indices proposed to date, including the most popular *HHI*, lack this value-validity property (P5), the new concentration index *C*_*K*_ is introduced. Besides having this additional property, *C*_*K*_ shares properties (P1-P4) with other indices, including *HHI*.

One desirable property of any summary measure is its simplicity and meaningfulness. The *C*_*K*_ is certainly simple to compute and, as discussed, has meaningful interpretations. Computationally, *C*_*K*_ simply equals the largest market share (divided by 1), plus the second largest market share divided by 2, plus the third divided by 3, and so forth for all the *n* firms within the market or industry. The fact that the computation of *C*_*K*_ requires the market shares to be ordered from the largest to the smallest as in (1) is not a real disadvantage since market-share data are typically reported in that format.

From the definition of *C*_*K*_ in (17), it is clear that when the number of firms *n* is large, the weights (1/*i*) assigned to the different market shares (*p*_*i*_) become sufficiently small such that the very small *p*_*i*_‘s can effectively be ignored from the computation of *C*_*K*_. This is really a practical advantage of *C*_*K*_ (as it also is with *HHI*) since market-share data are typically reported for the larger firms while the very small firms are grouped into an “others” category_._

Reported results from research involving market concentration seem to be increasingly based on the *HHI*. The empirically determined relationship in (25) can then conveniently be used to derive the corresponding values of *C*_*K*_ to a high degree of accuracy. Thus, valid concentration comparisons can be based on *C*_*K*_.
